# Expression and Function of CD44 in Epithelial Ovarian Carcinoma

**DOI:** 10.3390/biom5043051

**Published:** 2015-11-11

**Authors:** Joelle D. Sacks, Maria V. Barbolina

**Affiliations:** Department of Biopharmaceutical Sciences, University of Illinois at Chicago, 833 S Wood Street, 335 College of Pharmacy Building, Chicago, IL 60612, USA; E-Mail: jsacks3@uic.edu

**Keywords:** CD44, epithelial ovarian carcinoma, hyaluronan, adhesion, metastasis, cancer stem cells

## Abstract

CD44, a cell surface glycoprotein, has been increasingly implicated in the pathogenesis and progression of epithelial ovarian cancer, the deadliest gynecologic malignancy in women. Here, we review recent reports on the expression and function of CD44 in epithelial ovarian carcinoma. Further functional data for CD44 in peritoneal adhesion and metastatic progression and its association with stem cells is highlighted. Recent studies utilizing CD44 for therapeutic targeting are also discussed.

## 1. Introduction

**Figure 1:**
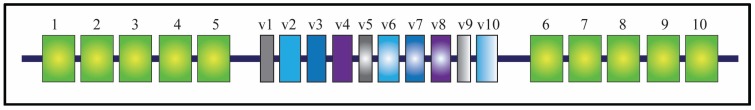
CD44 mRNA Transcripts. CD44 pre-mRNA is encoded by 20 exons, 10 of which can be regulated by alternative splicing of variant exons. The smallest CD44 isoform, which is known as CD44 standard (CD44s), includes only standard exons 1–5 and 6–10. CD44 variant isoforms include the addition of a combination of variant exons v1–v10 [6].

Members of the CD44 family of transmembrane glycoproteins (also referred to as HCAM, Pgp-1, Hermes antigen, and lymphocyte homing receptor) belong to a class of cell adhesion receptors involved in a variety of cellular processes including the regulation of growth, survival, differentiation, and motility [1]. The amino-terminal globular protein domain of CD44 is encoded by the first five non-variable exons of the CD44 gene and contains motifs that function as docking sites for several components of the extracellular matrix including hyaluronan (HA), which CD44 binds with a particularly high affinity, as well as collagen, laminin, and fibronectin. Exons 6–15 encode variant exons v1–v10, which are either completely excluded in the smallest CD44 isoform, CD44s, or included in various combinations in the extracellular domain leading to CD44 variant isoforms (Figure 1). Up to ten different CD44 isoforms have been reported due to differential splicing of the 10 variant exons [2,3]. Finally, the c-terminus cytoplasmic-tail region contains motifs important for binding intracellular protein partners, as well as phosphorylation sites, which facilitate association of CD44 with the cytoskeleton responsible for the associated phenotypes. The CD44 family is further compounded by several post-translation modifications including *N*- and *O*-glycosylation [4]. There are many reports about the physiological roles of CD44 and its activity in various diseases, but a detailed understanding of molecular mechanisms is mostly lacking [5].

CD44 is frequently expressed in a wide variety of epithelial malignancies, including ovarian cancer. Epithelial ovarian carcinoma ranks fifth in fatal tumors among women, accounting for more deaths than any other female gynecological cancer [7]. Ovarian cancer has a distinctive biology and behavior at the clinical, cellular, and molecular levels. While metastases can occur through lymphatics [8] or through blood vessels [9], most frequently, cancer cells are shed off of the primary tumor and implant on the peritoneal surface, forming numerous nodules throughout the abdominal cavity [10,11,12]. Although 70% of ovarian cancers will respond to a combination of platinum- and taxane-based chemotherapy administered after surgery, but most cases frequently recur and become resistant to treatments. In order to more strategically design therapeutics for ovarian cancer, it is crucial to not only identify, but fully characterize targets, like CD44, which play a role in recurrence, metastasis, and drug resistance in ovarian cancer. This review highlights recent advances in our understanding of the role CD44 plays in the biology of ovarian cancer.

## 2. CD44 Expression

CD44 is expressed in the majority of epithelial ovarian carcinomas [13,14,15]. While CD44 is low to absent in normal ovarian epithelial cells [16,17], CD44 has been identified as a potential marker for normal stem-like epithelial cells in the distal end of the fallopian tube [18]. Both CD44s and CD44 variants play complex roles in tumor progression and metastasis [19,20,21,22]. Expression of CD44 and specific isoforms in epithelial ovarian carcinoma has remained a controversial topic, as highlighted in a 2011 report, which reviewed some of the conflicting data on CD44 expression and its correlation with metastasis and survival outcome [23]. The authors pointed out that several studies have suggested that patients with CD44 positive tumors have a significantly shorter disease-free survival than patients with CD44 negative tumors [15,24,25], while in contrast, other studies have demonstrated that high CD44s expression is associated with improved ovarian cancer outcome [16,26,27]. Additionally, other studies have found no association between CD44s or CD44 variant expression with ovarian cancer metastasis or survival outcome [17,28,29]. They concluded that differences between the studies could be attributed to technical factors, including the use of different antibodies and detection methods. Moreover, the cohorts of ovarian cancer patients examined in the various studies were highly heterogeneous and composed of patients with variable tumor types, stages, and courses of therapy. An expanded summary of the published data on the contradiction of CD44 expression in ovarian cancer tissues is included in Table 1.

Recently published studies are in support of CD44 association with unfavorable prognosis in epithelial ovarian carcinoma [13,14,30,31,32]. Specifically, a recent study conducted using patient-matched primary tumor samples showed higher expression levels of CD44 in metastatic and recurrent tumor tissues when compared with its primary counterparts and a significant correlation between CD44 expression and decreased disease-free, as well as overall survival [32]. This group also reported overexpression of CD44 in the recurrent tumors generated using a xenograft mouse model exposed to paclitaxel treatment, providing evidence that CD44 may directly participate in chemoresistance; although, the underlying mechanisms were not MDR1-dependent. Similar results were reported by Zhang, *et al.* showing that CD44 expression associated with high-grade and advanced-stage ovarian carcinoma [33]. In further support, another group demonstrated that CD44 expression was also significantly increased in patients with metastasis [30]. However, no significant differences in CD44 expression was observed between paired primary and peritoneal metastases [30]. This contradiction to Gao *et al.* could be attributed similarly to the highly heterogeneous collection of ovarian cancer patients analyzed in these studies.

Recently, several studies have also focused on investigating the expression of CD44v6 to address its association with tumor progression, metastasis, and recurrence [34,35,36,37,38]. In ovarian cancer, though, the results from these studies have also not been consistent. Sakai, *et al.* reported no significant differences in expression of CD44v6 in matched primary and metastatic epithelial ovarian cancer lesions [39]. Similarly, no significant differences in CD44v6 expression was observed between benign, malignant primary tumors, metastatic lesions, and effusions cells [34]. These reports are in agreement with previously published findings, which did not show any correlation between CD44v6 expression and the risk of development of metastasis in ovarian carcinoma [40,41]. In contrast, another study reported an association between up-regulation of CD44v6 and ovarian carcinoma progression [42]. This is consistent with more recently published studies demonstrating that CD44v6 expression was upregulated in tumor tissues from recurrent disease and abdominal cavity metastases [37]. This compared similarly to Zhou, *et al.* and their reported data showing expression of CD44v6 associated with histological type, FIGO stage and histological grade, and 5-year survival suggesting CD44v6 as an important molecular marker for poor prognosis [36]. In 2014, Wang, *et al.* also described an association between CD44v6 expression and poor prognosis in patients with ovarian serous carcinoma [38]. Furthermore, a 2015 report recently identified that disseminated tumors in the pelvic peritoneum are highly enriched in CD44v6-positive cancer cells [43]. Differences between the studies could be attributed to technical factors including the use of different antibodies and detection methods. Use of qRT-PCR and FACS analysis, in addition to immunohistochemistry, has provided new quantitative data in support of expression of CD44v6 and its association with EOC progression, metastasis, and relapse [36]. These findings indicate that CD44v6 might be an important and useful marker for a poor prognosis in women with ovarian carcinoma, as well as a potentially effective therapeutic target for preventing and treating the recurrence of peritoneal metastasis.

**Table 1:** CD44 Expression in Epithelial Ovarian Cancer Tissues.

Sample Type	Method(s) & Antibodies	Results	Reference
31 epithelial ovarian tumors (24 serous, 5 endometrioid, 2 clear cell)	RT-PCR, IHC pan-CD44 (clone 25.32) CD44v3 (clone BBA11) CD44v4 (clone 11.10) CD44v6 (clone 11.9) CD44v9 (clone 11.24)	High expression of CD44s and CD44v9 in EOC; lack of association between CD44v and prognosis	[17]
21 epithelial ovarian tumors (13 serous, 2 endometrioid, 6 mucinous)	RT-PCR	CD44s amplified in all specimens; CD44R1/s ratio higher in metastases than in primary tumors	[44]
22 epithelial ovarian tumors (9 serous, 8 mucinous, 5 undifferentiated)	IHC CD44v5 (clone VFF-8) CD44v6 (clone VFF-7) CD44v7-8 (clone VFF-17)	Low CD44v5, v6, and v7-8 expression	[45]
44 epithelial ovarian tumors (26 serous, 12 mucinous, 6 other)	IHC CD44 v3-10 (clone 1.1ASML)	Association between increased CD44v expression and reduced survival	[24]
11 ovarian tumors (6 serous, 1 endometrioid, 2 clear cell, 1 yolk sac, 1 dysgerminoma)	IHC CD44v1-10 CD44v6 (clone 2F10) CD44v9	Expression of CD44v6 involved in nodal metastasis	[42]
76 epithelial ovarian tumors (20 benign, 36 borderline; 20 carcinoma: 10 serous, 10 mucinous)	IHC pan-CD44 (clone 2C5) CD44v3 (clone 3G5) CD44v6 (clone 2F10)	CD44 isoforms in ovarian borderline tumors of littlediagnostic and prognostic value	[46]
43 epithelial ovarian tumors (29 serous, 6 mucinous, 5 clear cell, 3 endometrioid)	IHC CD44v6 (clone BBA13)	CD44v6 expression greater in recurrent tumors compared to primary	[47]
56 epithelial ovarian tumors (38 serous, 6 poorly differentiated, 5 mucinous, 3 clear cell, 2 mixed Müllerian, 2 endometrioid	IHC pan-CD44 (clone SFF-304)	CD44 expression was significantly associated with poorer overall survival	[15]
115 epithelial ovarian tumors carcinomas (50 serous, 19 mucinous, 16 endometrioid, 30 clear cell), 32 low malignant potential (LMP) tumors (3 serous, 29 mucinous), 53 cystadenomas (14 serous, 39 mucinous)	RT-PCR, IHC pan-CD44 (clone DF1485) CD44v3 (clone 3G5) CD44v6 (clone 2F10)	CD44 expression upregulated during ovarian carcinoma development, but down-regulated during progression; loss of CD44v3 expression predicts poor prognosis	[13]
56 epithelial ovarian tumors (40 serous, 5 mucinous, 3 endometrioid, 5 clear cell, 3 undifferentiated)	IHC CD44v6 (clone 2F10)	No significant change in CD44v6 expression in primary and matched metastatic sites	[39]
50 epithelial ovarian tumors (25 serous, 10 mucinous, 9 endometrioid, 6 other)	IHC pan-CD44 (clone SFF-2) CD44v5 (clone VFF-8) CD44v6 (clone VFF-7) CD44v7-8 (clone VFF-9) CD44v10 (clone VFF-14)	High expression of CD44v5; CD44v5 and v6 associated with intraperitoneal implantation	[48]
28 epithelial ovarian tumors (all serous; 1 well-differentiated, 12 moderately differentiated, 15 poorly differentiated)	IHC pan-CD44 CD44v5 (clone VFF-8) CD44v6 (clone VFF-7) CD44v7-8 (clone VFF-17)	Increased expression of CD44s and CD44v5 in stage III compared with stage I tumors.	[25]
101 epithelial ovarian tumors (45 serous, 9 endometroid, 5 clear cell 5 mucinous; 18 LMP: 13 serous, 4 mucinous-GI type, 1 endometrioid)	IHC pan-CD44 (clone A3D8)	Decreased expression of CD44s associated with shortened survival	[26]
142 epithelial ovarian tumors (93 serous, 15 mucinous, 14 endometrioid, 21 LMP)	IHC pan-CD44 (clone 2C5) CD44v3 (clone 3G5) CD44v4 (ATCC 257-HB) CD44v5 (clone VFF-8) CD44v6 (ATCC 256-HB) CD44v9 (ATCC 258-HB) CD44v10 (clone VFF-14)	Expression of CD44s, CD44v4, CD44v6, and CD44v10 in primary EOC associated with increased survival	[27]
307 epithelial ovarian tumors (109 serous, 31 mucinous, 82 endometrioid, 32 clear cell, 53 other)	IHC pan-CD44(clones 2C5, DF1485)	High CD44 expression associated with favorable prognosis	[16]
83 epithelial ovarian tumors (31 serous, 4 mucinous, 3 mixed, 3 clear cell, 7 poorly differentiated; 14 borderline, 21 benign)	IHC pan-CD44 (clone DF1485)	No correlation between CD44 and survival	[49]
158 epithelial ovarian tumors (95 malignant serous, 23 benign, 40 borderline)	IHC pan-CD44 (clone DF1485) CD44v6 (clone VFF-7)	Overexpression of CD44 associated with high grade, advanced stage, and low overall survival rate	[14]
57 epithelial ovarian tumors (all serous)	IHC CD44v6 (VFF-18)	CD44 expression higher in borderline and carcinomas compared to benign, but no significant correlation with clinicopathological factors	[35]
62 epithelial ovarian tumors (45 serous, 5 mucinous, 8 endometrioid, 4 undifferentiated)	IHC CD44v6 (clone VFF-7)	CD44v6 an important molecular marker for poor prognosis	[36]
65 epithelial ovarian tumors (all serous)	IHC pan-CD44 (clone IM7) CD44v6 (clone VFF-18)	CD44v6 expression associated with progression, metastasis, and relapse	[37]
72 epithelial ovarian tumors (all serous: 14 well-differentiated, 36 moderately differentiated, 22 poorly differentiated)	IHC pan-CD44 (clone DF1485)	No change in expression between primary tumors and peritoneal metastases; Increased CD44 expression in patients with metastases	[30]
483 primary epithelial ovarian tumors (393 serous, 35 endometrioid, 16 clear cell, 11 transitional cell, 28 other) 127 paired primary and recurrent tumors	IHC pan-CD44(clone 156-3C11)	CD44 expression associated with high grade and advanced stage carcinoma, but not with overall survival; no statistical difference in CD44 expression between primary and recurrent disease	[33]
26 paired primary, metastatic, and recurrent epithelial ovarian tumors from well-characterized late-stage ovarian cancer patients	IHC pan-CD44(clone 156-3C11)	Significant association between CD44 expression and both disease-free and overall survival	[32]

## 3. Post-Translational Modifications of CD44

Glycoproteins, including CD44, carry various sugar moieties on their extracellular domains [6,50]. These glycoconjugates are involved in cell growth and differentiation and are important components of the cell membrane [51]. CD44 is highly glycosylated with multiple *N*- and *O*-linked sites and alternative splicing of CD44 isoforms provides new oligosaccharide attachment sites, which result in potentially functionally significant glycosylation changes that have been implicated in the regulation of CD44-mediated cell binding to HA [4]. These glycans may include, but are not limited to, galactosamine and glucosamine moieties, as well as mannose, glucose, fucose, and sialic acid [52]. Alternatively, CD44 can have additional post-translational modifications including the attachment of chondroitin sulfate and heparin sulfate [6]. A few studies have investigated the role of CD44 glycosylation and HA adhesion in ovarian carcinoma cells. One study concluded that cell membrane glycosylation mediates the adhesion, migration, and invasion of ovarian carcinoma cells [53]. Another study demonstrated that the glycosylation, but not the spliced variant content of CD44, affects the adhesive properties of ovarian tumor cells [54]. The nature of the specific attached oligosaccharides is only just beginning to be explored. Lewis y antigen, an oligosaccharide with two fucoses, is frequently overexpressed in epithelial ovarian cancer. CD44 is abundant in α-l-fucose, and is an important α1, 2-fucose antigen-containing protein on the surface of cells [55]. Recently, it was shown that Lewis y antigen, as a structure within the CD44 molecule, strengthens CD44-mediated adhesion to HA and the spreading of ovarian cancer cells [56]. This group then followed up reporting significant correlations between expression of Lewis y antigen and CD44 in tissues from ovarian cancer patients, where overexpression of Lewis y and CD44 antigen were strong predictive risk factors for chemotherapeutic drug resistance [57]. Similar results were found in a subsequent clinical study of patients with chemotherapy resistant or sensitive EOC where high expression of both Lewis y and CD44 correlated with chemotherapy resistance [58]. Further studies might offer insight into whether these two molecules regulate each other or are co-regulated together for functional enhancement of CD44 signaling.

## 4. CD44 and Stemness

One model that has arisen to explain tumor recurrence in ovarian cancer is the “Cancer Stem Cell Hypothesis” [59]. These cells are also often characterized as chemoresistant, because of their stem cell qualities. The search for ovarian CSCs has largely been based on the exploitation of properties that are associated with “stemness” in other systems, particularly the expression of specific “stemness” markers on the cell surface. CD44 has been frequently implicated as a marker of both somatic and CSCs [60]. Interestingly, a 2012 study identified CD44^+^ stem-like epithelial cells in the human fallopian tube concentrated at the fimbria. These cells generated spheres *in vitro* that contained all functional fallopian tubal epithelia cell types [18]. This is important as the cell of origin for serous ovarian cancer remains under debate since a gene profiling study showed that the gene expression profile of high grade serous ovarian carcinoma is more closely related to fallopian tube epithelium than to ovarian surface epithelium supporting the view that serous tumors develop from the fallopian tube [61]. Studies on the properties of these “stem” cells in ovarian cancer using an Aldefluor assay to isolate ALDH1-bright (ALDH1^br^) cells from epithelial ovarian cancer cell lines, revealed greater stem-like properties, an enrichment in CD44, and association with chemoresistance and poor clinical outcome [62]. Other reports have showed that CD44^+^ status along with CD117^+^ is a marker of ovarian cancer-initiating cells [63], while when CD44^+^CD117^+^cells were compared using two-dimensional (2D) culture *versus* a three-dimensional (3D) culture system, CD44^+^CD117^+^ cells possessed not only the CSC properties, but also exhibited increased chemoresistance in 3D compared to 2D culture, suggesting a more relevant model for studying CSC response to anticancer drugs [64,65]. Furthermore, fresh primary high-grade serous ovarian carcinoma biopsies cultured under serum-free conditions produced floating spheres, which overexpressed stem cell genes and CSC markers, including CD44 [66]. In order to further understand the effects of CD44 in ovarian cancer stem cells, an anti-CD44 monoclonal antibody A3D8 was employed to investigate its effects and mechanisms on the proliferation and apoptosis of sphere-forming cells in the human ovarian cancer cell line SKOV-3. Results showed that A3D8 inhibited proliferation of sphere-forming cells and induced apoptosis in these cells causing S-phase arrest and deregulation of the cell cycle [67]. Moreover, transfection of CD44^+^⁄CD117^+^ cells enriched from human primary ovarian tumor tissues with miR-199a, significantly decreased CD44 mRNA and protein expression while significantly affecting cell cycle regulation, suppressing proliferation, and increasing chemosensitivity of ovarian CICs. Luciferase reporter gene assays confirmed that miR-199a targets CD44 via a miR-199a-binding site in the 3′-UTR [68]. These observations suggest that CD44 and miR-199a play a very important role in regulating the cancer stem cell phenotype of CIC in ovarian cancer, supporting the hypothesis that CD44 may provide a novel therapeutic target for ovarian cancer. Interestingly, Tjhay *et al.* showed that CD44v6 expression demarcates a highly tumorigenic ovarian CSC population with peritoneal metastatic potential reporting that CD44v6-positive cells possess the potential to serve as metastasis-initiating cells [43]. Thus, CD44v6, more specifically, may be a potential molecular therapeutic target for eliminating CSCs and metastasis-initiating cells in epithelial ovarian cancer.

## 5. CD44 in Peritoneal Adhesion

Previous research has shown that human ovarian tumor cells can bind HA via membrane CD44 [69]. CD44 has also been shown to mediate ovarian carcinoma cell adhesion to peritoneal mesothelial cells [70], while other *in vivo* studies have suggested that CD44s is required for human ovarian cancer cell adhesion to mesothelial cell surface HA [19]. Therefore, it has been established that ovarian cancer cell adhesion to mesothelial cell monolayers is mediated, at least in part, by the interaction between HA and CD44 [56]. Moreover, dependence of CD44 on versican, a large HA-binding proteoglycan, has been shown in ovarian cancer where HA and versican form a pericellular matrix around CD44-expressing ovarian cancer cells that further promotes their motility and invasion *in vitro* [71]. Studies in our lab demonstrated that versican is involved in facilitating both ovarian cancer cell and spheroid adhesion to mesothelial cell monolayers. Likewise, both spheroids and cells with reduced expression of versican demonstrated significantly impaired ability to generate peritoneal tumors suggesting that versican functions in regulating the development of peritoneal metastasis originating from single cells and spheroids. This further supports a role for CD44 together with HA and versican in a number of key steps needed for ovarian cancer metastasis [72]. However, mechanisms that induce expression of CD44 in ovarian cancer are poorly understood. A recent study found that expression of CD44 was induced by the transcription factor DLX4 via enhanced activity of NF-κB stimulated by inflammatory cytokine IL-1β, a transcriptional target of DLX4. High expression of DLX4 is associated with reduced survival of ovarian cancer patients and found to stimulate attachment of ovarian tumor cells to peritoneal mesothelial cells *in vitro* and increase the numbers of peritoneal implants in xenograft models [73]. DLX4, therefore, might contribute to poor outcomes in ovarian cancer, in part, by promoting peritoneal implantation of tumor cells via enhanced CD44 expression and tumor-mesothelial cell interactions in an NF-κB-dependent manner. Further investigation into the cross-talk between CD44 and inflammatory signaling in ovarian cancer will hopefully lead to a better understanding of more effective focal points for therapeutic intervention. Moreover, post-transcriptional regulation of CD44 by multiple non-coding RNAs (ncRNA) including miRNAs and lncRNAs have been described in hepatocellular carcinoma [74], osteosarcoma [75], prostate cancer [76,77], and gastric cancer [78,79]. Although miR-199a was reported to target CD44 in ovarian cancer [68], additional studies are needed to further characterize these CD44 regulatory mechanisms in epithelial ovarian cancer.

## 6. Therapeutic Targeting of CD44

In several types of tumors, CD44 together with other cell surface markers characterizes cancer stem cell populations. Mechanistically, CD44 proteins act as receptors for hyaluronan, co-receptors for receptor tyrosine kinases or G-protein-coupled receptors, or provide a target for metalloproteinases [80]. For all these reasons, targeting CD44 may be a successful approach in cancer therapy. As CD44 is the main receptor for hyaluronan, and the hyaluronan binding domain exists in all CD44 isoforms, much effort has focused in blocking the CD44-HA interaction based on abundant evidence that the CD44-HA interaction is involved in tumor progression [81,82]. It has been shown that the interference with the binding of CD44 expressed on tumor cells to HA using either the soluble CD44 ectodomain as a competitor or antibodies that specifically block the binding of HA to CD44, impaired tumor growth and metastasis in breast cancer cells [83,84], while HA oligosaccharides had similar effects in inhibiting tumor growth *in vivo* [84]. Pan-CD44 monoclonal antibodies have been shown to reduce tumor growth, metastasis and post-radiation recurrence of pancreatic xenograft tumors [85], while have also been reported to drastically decrease the leukemic population in mice transplanted with human acute myeloid leukemic stem cells, as well [86]. Furthermore, isoform-specific antibodies have been reported, including those against CD44v6, where a radiolabeled CD44v6 monoclonal antibody showed selective tumor targeting and high tumor uptake in a nude mouse squamous cell carcinoma xenograft [87]. In addition to antibody-based targeting of CD44, other strategies have been employed using DNA aptamers targeting CD44v10 in breast cancer [88] and peptides mimicking CD44v6 for blocking the coreceptor function of CD44v6 for c-Met and VEGFR-2 in endothelial cells thereby impeding angiogenesis [89]. In an effort to develop new and effective treatment strategies for advanced-stage ovarian cancer patients, CD44 is also being explored as a therapeutic target. Instead of interfering with the function of CD44 proteins, these studies have aimed at inhibiting CD44 expression in tumor cells. One recent report tested a dendrimer-based drug delivery system for carrying paclitaxel and siRNA targeted to CD44 mRNA while another constructed PLGA nanoparticles with short hairpin RNA (shRNA) against focal adhesion kinase (FAK) and CD44 for enhancing antitumor effects in an ovarian cancer mouse xenograph [90,91]. Both showed a high therapeutic potential for combinatorial treatment of ovarian carcinoma using novel nanoscale drug delivery systems. Although only few approaches have made it as far as clinical trials, the scientific progress in the last few years suggests strong prospects for anti-CD44 therapies.

## 7. Conclusions

For quite some time, the role of CD44 in ovarian cancer progression and metastasis has remained unclear. However, many lines of evidence indicate that CD44 organizes a signaling platform by which cancer cells survive and grow in addition to seeding metastases. The published reports highlighted in this review provide further evidence that CD44 is one of the main players in ovarian tumor growth and metastasis. Moreover, a fair amount of data now supports CD44 as a genuine marker of ovarian CSCs. However, it must be noted that the detection of CD44 on EOC CSCs was performed with antibodies that recognize all CD44 isoforms. Since CD44s is expressed ubiquitously in tissues, there is a critical need to define which CD44-specific isoforms are present on these CSCs. Greater understanding of the functions of CD44 isoforms at the molecular level and identification of specific CD44 isoforms on CSCs will, then, allow new strategies to be directed more discerningly against tumor cells. Finally, there is a hypothesis that CD44 function varies during different stages of tumor growth, from initiation to formation of metastases [92], which has yet to be explored in the context of epithelial ovarian cancer. Based on current understanding, CD44 remains promising as a therapeutic target in ovarian cancer and, thus, warrants further evaluation.
